# Development of health emergency response capability evaluation framework for primary health institutions in metropolis: based on Delphi method and analytic hierarchy process

**DOI:** 10.3389/fpubh.2025.1577853

**Published:** 2025-07-23

**Authors:** Qian Li, Hongjie Yu, Fan Cheng, Haidong Kuang, Xiaoqiong Zhang, Yuqing Shao, Xiaoxue Ma, Jingyu Li, Yan Li, Yanhong Zhu, Yipeng Lv

**Affiliations:** ^1^School of Public Health, School of Medicine, Shanghai Jiao Tong University, Shanghai, China; ^2^Jiading District Center for Disease Control and Prevention, Shanghai, China; ^3^Shanghai Engineering Research Center of Tooth Restoration and Regeneration, Tongji Research Institute of Stomatology, Department of Prosthodontics, Stomatological Hospital and Dental School, Tongji University, Shanghai, China; ^4^Yichuan Community Health Service Center, Shanghai, China; ^5^Shanghai General Hospital, Shanghai, China; ^6^Key Laboratory of Urban Complex Risk Control and Resilience Governance, Shanghai Emergency Management, Shanghai Jiao Tong University, Shanghai, China

**Keywords:** emergency response capacity, primary health institutions, metropolis, Delphi method, analytic hierarchy process

## Abstract

**Background:**

Primary health institutions, as the “first on-site responders,” play a crucial role in responding to health emergencies. However, there are few studies on the systematic assessment of their emergency response capabilities. We aimed to develop a health emergency response capability evaluation framework for primary health institutions to assess the resuscitation capacity in metropolis.

**Methods:**

In the first stage, we collected preliminary indicators through literature and government documents concerning the primary health evaluation. Afterward, we utilized the Delphi method to consult 15 frontline health emergency response practitioners, health management officials, and research experts. After two rounds of questionnaire consultations, participants scored the importance and feasibility of all indicators. Subsequently, we employed the analytic hierarchy process (AHP) to determine the weights assigned to each indicator and construct the framework of health emergency response capability evaluation for primary health institutions.

**Results:**

We developed a framework for evaluating the health emergency response capabilities of primary health institutions in metropolis, comprising 3 first-level indicators, 11 s-level indicators, and 37 third-level indicators. In both rounds of consultation, experts provided a unanimous positive consensus, with a 100% agreement rate. The authority coefficient was 0.92 for both rounds, and the *p*-value of Kendall’s W was statistically significant (<0.001). Furthermore, compared to the first round, the level of coordination among experts improved in the second round. In the process of judging matrices, the consistency ratios (CRs) of indicators at all levels were less than 0.1. For first-level indicators, including “prevention and monitoring,” “resource reserve and system construction,” and “emergency response and summarization,” the respective weight values were 0.286, 0.335, and 0.379, respectively.

**Conclusion:**

This study developed a framework for evaluating the health emergency response capabilities of primary health institutions in metropolis. This framework can help improve the evaluation systems for emergency response capacity in primary health institutions in China’s metropolis.

## 1 Introduction

Public health emergencies have significantly increased with the development of societies and increased human activities. For example, between November 1, 2002, and July 31, 2003, the severe acute respiratory syndrome (SARS) affected 8,096 cases and led to 774 deaths (1). In 2009, influenza A (H1N1) became the first 21st-century flu pandemic, leading to 18,449 deaths across 214 countries (2). From 2014 to 2016, the Ebola epidemic spread to West Africa, affecting 28,652 patients and leading to 11,325 deaths (3). From late 2019 to December 26, 2020, COVID-19 led to 80,500,000 confirmed cases and 1,700,000 deaths (4). Additionally, between May 13, 2022, and November 2022, the monkeypox virus affected over 78,000 patients across 100 countries (5). These crises necessitate a robust scientific framework for public health emergency response and enhanced capacity to face public health emergencies.

The evaluation of health emergency response capacity entails a systematic analysis of the comprehensiveness and coordination of emergency response elements. This evaluation serves several purposes, helping assess the proficiency in responding to public health emergencies and understanding the emergency response requirements of healthcare facilities at all levels. In addition, it can guide subsequent health resource allocation. It enhances the health emergency response system and bolsters the capability to address public health emergencies (6). The significance of assessing institutional vulnerability and capacity has also been emphasized in the “Sendai Framework for Disaster Risk Reduction 2015–2030,” proposed by the United Nations Office for Disaster Risk Reduction (UNDRR) (7). Different states have developed various tools to evaluate their health emergency response capacity. In 2013, Centers for Disease Control and Prevention, together with state health officials in the United States, developed the “National Health Security Preparedness Index (NHSPI)” to evaluate the readiness of states to manage health emergencies (8). Similarly, in 2012, the WHO Regional Office for Europe issued the “Toolkit for Assessing Health System Capacity for Crisis Management,” designed to assess European Union member states’ response to public health emergencies and enhance their preparedness (9). Furthermore, in 2013, the Emergency Response Office of the National Health and Family Planning Commission of China developed “The Health Emergency Response Capacity Assessment Questionnaire” for provincial and municipal health administrations. This questionnaire serves as a critical tool for governments to evaluate health emergency response capacity in their regions (10). The development of evaluation systems for health emergency response capacity has continued, with ongoing updates. The majority of current health emergency response evaluation systems focus on national or regional levels. They comprehensively measure regional resilience, which includes different aspects, such as emergency response coordination, communication, and material stockpiling. Nonetheless, there are few evaluation systems tailored to primary health institutions. The absence of systematic capacity evaluations obscures their emergency roles and operational mandates, significantly constraining their early-stage response functions during public health emergencies. This discrepancy between existing capabilities and operational demands underscores the urgent need to develop institution-specific health emergency response assessment frameworks.

Primary health institutions, as part of the primary healthcare system, aim to deliver essential healthcare services to individuals and families. They play a pivotal role in achieving the objective of “Primary Health Care for All” as outlined in the Declaration of Alma-Ata (11). Examples of such institutions include the National Health Service General Practitioner system in the United Kingdom (12), Singapore polyclinics (13), and community health service centers in China (14), all of which provide daily healthcare services to their respective communities. Currently, primary health institutions play a crucial role in responding to health emergencies in various countries. For instance, during the COVID-19 pandemic, community health service centers in China actively managed the sources of infection, blocked the transmission pathway, and safeguarded susceptible populations (15). However, some scholars mentioned that available primary health institutions primarily engage in auxiliary emergency response, overlooking their potential as the nearest healthcare units to their communities (16). This is largely due to the absence of a systematic assessment of their emergency response capabilities. Consequently, it significantly hampers the emergency response function of primary health institutions during the early stage of emergency conditions. In 2022, China had 36,448 community health service centers (17). Assessing the emergency response capacity of these centers and fully mobilizing their resources and capabilities during the initial stages of crises holds the potential to enhance their response efficacy.

In China’s metropolis characterized by high population density and significant mobility, the frequency of public health emergencies increases while control challenges become more complex, thereby imposing stricter requirements on the emergency response capabilities of primary health institutions. Furthermore, metropolis play a pivotal role in formulating and implementing public health policies, with their emergency response experiences and lessons providing valuable references for other regions. The evaluation system for emergency response in primary health institutions in metropolis could offer replicable and scalable models for developing health emergency capacities in primary health institutions across other cities or regions. Therefore, this study focused on primary health institutions in China’s metropolis as the research subject. Current research on health emergency capacity assessment primarily focused on national or regional levels, leaving a significant research gap in evaluation tools for primary health institutions. Given the critical role of primary health institutions in early-stage management of public health emergencies, this study employed the Delphi method and Analytic Hierarchy Process (AHP) to construct a comprehensive evaluation system for health emergency capabilities in metropolitan primary health institutions. The system will clarify capacity-building directions, provide scientific evidence for health resource allocation, and ultimately optimize the emergency response effectiveness of primary health institutions during public health emergencies.

## 2 Methods

**Figure 1:**
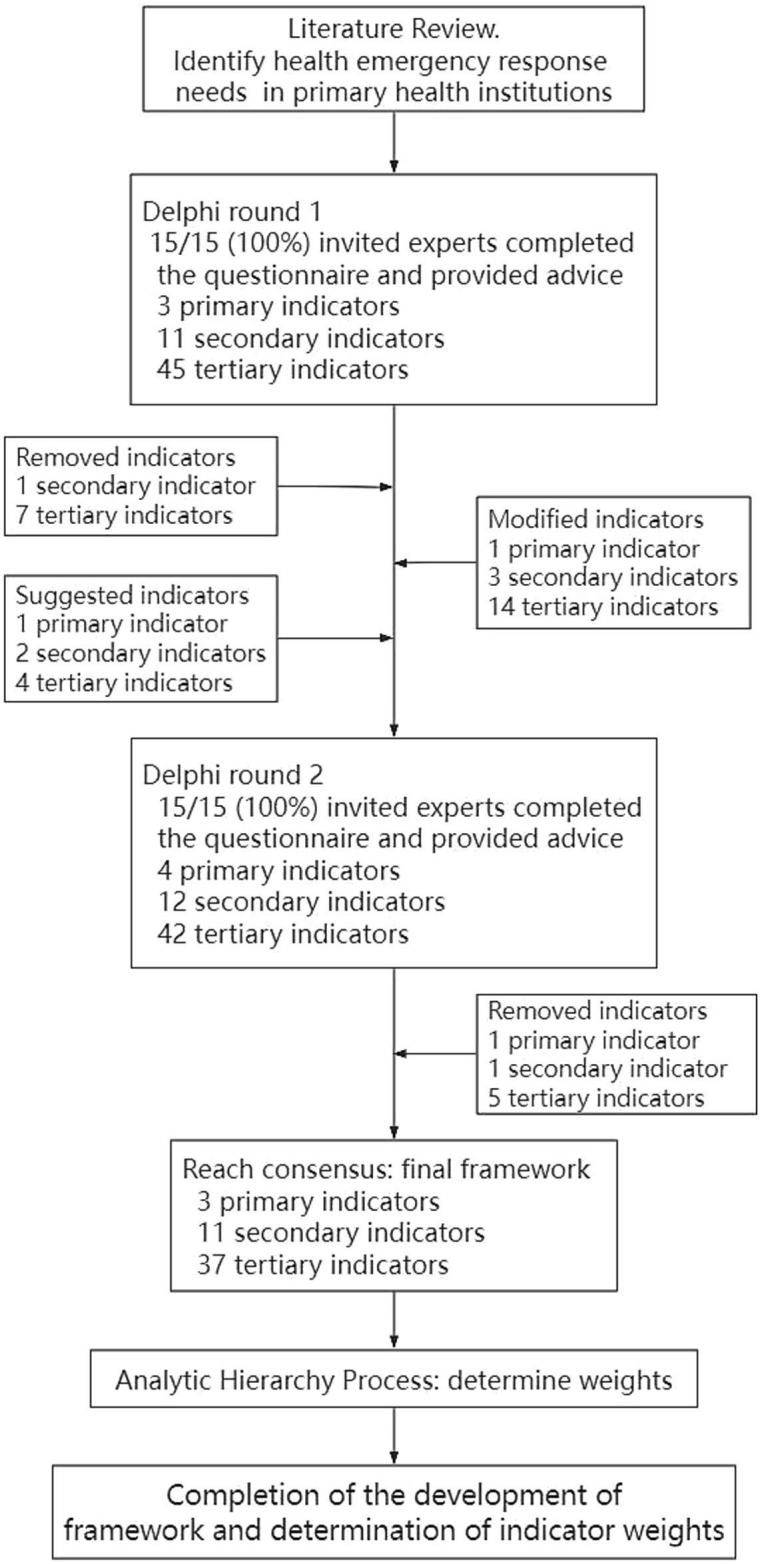
Flowchart showing the design of a framework to evaluate the health emergency response capability of primary health institutions.

This study utilized a combination of qualitative and quantitative analyses to evaluate the emergency response capacity of primary health institutions. The Delphi and AHP methods were applied to construct the framework of health emergency response capability evaluation for primary health institutions and determine the corresponding weights. The study flowchart is illustrated in Figure 1.

This study strictly adhered to the ethical principles of the Declaration of Helsinki. As the research data only involved experts’ basic characteristics and rating results without any personally identifiable information, the institutional review board approval was waived in accordance with relevant ethical guidelines.

### 2.1 Theoretical sources

This study adopted the prevention, preparedness, response, and recovery (PPRR) model as its primary reference, which categorizes the crisis management process into four distinct phases: prevention, preparedness, response, and recovery, and mirroring the evolution of crises (18).

The two ‘P’ stages within the PPRR model serve as the foundation and prerequisite for crisis management, aiming to mitigate the risk of crises. The two ‘R’ stages aim to minimize the harmful effects of public health emergencies and facilitate a timely return to normal conditions. The PPRR model adopts different coping strategies according to the stage of the crisis, rendering it scientific, comprehensive, and practical. Based on the PPRR model, this study divided the emergency response to public health emergencies into four phases: prevention, preparation, response, and recovery, establishing indicators on this basis.

### 2.2 Constructing the initial evaluation indicator base

To establish a scientific and rational evaluation index system for public health emergency response capacity in primary healthcare institutions, our indicator development process referenced two main categories of literature. First, we prioritized national policy documents including the “Guiding Opinions on Strengthening the Performance Evaluation of Primary Healthcare Institutions (for Trial Implementation)” released by the National Health Commission in 2020 (19) and the “Guidelines for Evaluating the Service Capability of Community Health Service Centers” issued in 2022 (20) to clarify the mandated emergency responsibilities regarding infectious disease reporting, nosocomial infection prevention, and two-way referral systems. Second, we systematically reviewed multi-level assessment tools such as the United States’ National Health Security Preparedness Index (NHSPI) (8), Europe’s Health Emergency Preparedness Self-Assessment Tool (HEPSA) (9), China’s “Health Emergency Capability Assessment Survey Questionnaire,” (10) followed by rigorous literature analysis and expert consultations to localize and screen appropriate indicators. Based on the PPRR model, to achieve structural balance in the primary indicators, the response and recovery phases were systematically integrated. The design encompassed three first-level indicators: “prevention and monitoring,” “resource reserve and system building,” and “response and summarization.” Subsequently, second-level and third-level indicators were sequentially and systematically screened and formulated.

### 2.3 Criteria for including experts

Fifteen experts in the field of health emergency response were enrolled, including personnel from governmental agencies, frontline workers, and researchers focusing on emergency response. No data related to the medical institutions of the experts were involved in this study; thus, the consent of their medical institutions was not needed. The inclusion criteria were as follows: (1) possessing more than 5 years of experience in community health management or related research, with a comprehensive understanding of the current state of primary health services; (2) being familiar with the rules and methods of response to pandemics; (3) being interested in and available for active participation in this study.

### 2.4 Delphi process

We electronically sent the expert consultation form to the chosen experts and reminded them to return it within the stipulated deadline. The indicator system underwent revisions, deletions, and additions, guided by the predetermined screening criteria and textual amendments from the experts. For indicators with divergent opinions, the research team anonymized and provided feedback on expert opinions to the panel, facilitating discussion and integration of viewpoints. A resolution was ultimately formed based on majority consensus, with modification proposals then circulated to relevant experts for final confirmation to ensure a conclusive agreement was reached. A consensus was achieved following two rounds of expert consultation, resulting in the establishment of a complete indicator system.

During each round of the Delphi process, experts were asked to evaluate the importance and feasibility of indicators at each level and provide suggestions for revisions. The importance of indicators was rated on a scale of 1 to 5, with higher scores representing greater importance. Similarly, the feasibility of each indicator was assessed on a scale of 1 to 5, with higher scores representing greater feasibility. Additionally, experts’ familiarity with the indicators was rated on a scale of 0.2 to 1 (0.2, 0.4, 0.6, 0.8, or 1), with higher values representing greater familiarity. The judgment criteria were based on four aspects: theoretical analysis, practical experience, previous studies from China or other countries, and intuitive feeling (see Supplementary File 1: Table S1).

### 2.5 Inclusion of indexes

This study adopted the boundary value method as the standard method to screen indicators and quantitatively identify the indicators with a relatively high degree of importance based on expert opinions. The specific screening methods were as follows:

- The threshold values for the percentage of perfect scores = mean–standard deviation. Scores above the threshold value were retained.
- The threshold values for the mean = mean–standard deviation. Scores above the threshold value were retained.
- The threshold values of the coefficient variation = mean + standard deviation. Scores below the threshold values were retained.

Indicators failing to meet the criteria for two or more of the three items were removed. Moreover, indicators lacking both importance and feasibility were excluded. When one of importance and feasibility failed to meet the criteria, adjustments or revisions were implemented based on the recommendations and insights from the expert cohort.

### 2.6 Weight determination of each indicator

The indicator weighting score sheet was distributed electronically to selected experts to prevent opinion bias from face-to-face interactions. The research team aggregated feedback for discussion, implementing an anonymized iterative deliberation process when conflicting opinions emerged, ultimately reaching resolutions through majority consensus. All modifications were returned to relevant experts for final validation to ensure conclusion consistency.

This study adopted the framework of health emergency response capability evaluation for primary health institutions as the target layer and constructed an AHP hierarchical model comprising a three-level evaluation index system. Subsequently, a pairwise comparison judgment matrix was formulated utilizing Saaty’s fundamental scales of 1–9^21^ to assess the relative importance of each index within the same hierarchical level. We then used the software to calculate the initial weights and combined weights, followed by conducting consistency tests. Using the group decision-making feature of the software, the group decision-making conclusion was derived by synthesizing the scores of multiple experts, thereby determining the weights for indicators at each level.

### 2.7 Data management and analysis

All data were analyzed using SPSS (SPSS, version 22.0). Whenever missing or incomplete questionnaire data were identified, we promptly contacted the respective expert for clarification and completion, ensuring the datasets were complete without any missing entries. The basic characteristics of the experts, including gender, age, title, education, and working years, are presented as numbers and proportions. The positive degree of experts (21) was expressed by the effective recovery rate of the questionnaire and typically exceeded 70% to affirm a high level of engagement. The authoritative coefficients (Cr) of the experts were determined by averaging the score of judgment basis (Ca) and the score of their acquaintance with the questions (Cs) (calculation formula: Cr = (Ca + Cs)/2). Generally, an authoritative coefficient greater than 0.7 indicates a high level of authority. The degree of concentration of expert opinion was described as the percentage of full scores and mean scores. The degree of dispersion of expert opinion was described as the coefficient variation (CV). A smaller variation coefficient indicated greater consensus among experts. The coordination of experts’ opinions was measured using Kendall’s coefficient of coordination, which ranges from 0 to 1, and higher values indicate better concordance. Yaahp 12.4 (yaahp software, Taiyuan, Shanxi, China) was used in the AHP process. The software can perform consistency tests, and CR < 0.1 suggests that the judgment matrix is in good consistency.

## 3 Results

### 3.1 Characteristics of experts

This study consisted of two rounds of Delphi, with 15 questionnaires distributed in each round. The effective return rate for both rounds was 100%, representing a high level of positive degree of experts. The expert panel comprised 15 consultants, all with over 5 years of experience in public health emergency response. Notably, 73.3% of the experts possessed more than 15 years of professional experience, and 60% held senior professional titles. The panelists were affiliated with diverse institutions, including community health service centers, Centers for Disease Control and Prevention(CDC), universities, and the National Health Commission. The basic characteristics of the experts are shown in Table 1.

**Table 1:** Demographic information of the experts (*n* = 15).

Basic information	*N*	Composition ratio (%)
Gender		
Male	9	60.0
Female	6	40.0
Age		
≤39	2	13.3
40 ~ 45	9	60.0
≥45	4	26.7
Title		
Senior	2	13.3
Deputy Senior	7	46.7
Intermediate	6	40.0
Education level		
PhD degree	3	20.0
Master’s Degree	2	13.3
Bachelor degree or below	10	66.7
Institutional contexts		
Community health service centers	7	46.7
Centers for Disease Control and Prevention	3	20.0
University	3	20.0
National Health Commission	2	13.3
Work experience		
≥21 years	6	40.0
15 ~ 20 years	5	33.3
≤15	4	26.7

### 3.2 Authoritative coefficient of experts

Based on the experts’ self-evaluation scores, the expert authority coefficients for the two rounds were 0.92 and 0.92, respectively. The experts’ self-evaluation scores were greater than 0.7, representing a high degree of expert authority. Detailed data can be found in Supplementary File 1: Table S2.

### 3.3 Concentration and variation in experts’ opinions

The concentration degree of expert opinions in this study was mainly represented by the mean scores (X), the coefficient variation (CV), and the full score ratio K (%). In the first round, for indicator importance, the X was 3.73 to 5.00, the CV was 0.00 to 0.45, and the K was 40.0 to 100.0%, for indicator feasibility, the X was 3.47 to 5.00, the CV was 0.00 to 0.45, and the K was 26.7 to 100.0%. In the second round, for indicator importance, the X was 3.20 to 5.00, CV was 0.00 to 0.53, and K was 26.7 to 100.0%, for indicator feasibility, the X was 3.33 to 5.00, the CV was 0.00 to 0.48, and the K was 26.7 to 100.0%.” The results show that, excluding the newly added indicators in the second round, the other indicators had higher mean scores, higher percentages of full scores, and lower variation coefficients, indicating a higher degree of consensus among experts in the second round of consultation. Detailed data can be found in Supplementary File 1: Table S3–S11 and Supplementary File 2: Table S12–S18.

### 3.4 Coordination of experts’ opinions

Kendall’s coefficient of concordance can be used to assess the agreement among experts. The value of *W* ranges from 0 to 1, where a higher value indicates stronger consensus among experts. In the first round of Delphi, the Kendall coefficients for importance and feasibility were 0.161 (X2 = 139.910, *p* = 0.00) and 0.197 (X2 = 171.584, *p* = 0.00), respectively. In the second round of Delphi, the Kendall coefficients W for importance and feasibility were 0.169 (X2 = 144.829, *p* = 0.00) and 0.199 (X2 = 170.095, *p* = 0.00), respectively. The *p*-values of Kendall’s coordination coefficients for the two rounds of expert consultation were <0.001 in this study, suggesting statistical significance. Moreover, Kendall’s coefficients observed in the second round of expert consultation were higher than those observed in the first round of expert consultation. This indicated that compared with the first round, the degree of coordination among experts was improved in the second round. In addition, the degree of coordination among experts in both rounds was satisfactory.

### 3.5 Establishment of the indicator system

We revised the indicators based on the indicator screening criteria and expert opinions. Based on the final consensus, the health emergency response capability evaluation framework for primary health institutions in metropolis included 3 first-level indicators, 11 s-level indicators, and 37 third-level indicators (Table 2). The finalized first-level indicators include “Prevention and Monitoring,” “Resource Reserve and System Building,” and “Emergency Response and Summarization,” covering the entire crisis management process. The specific explanation and scoring criteria for tertiary indicators can be found in Supplementary File 2: Table S18.

**Table 2:** The final evaluation framework of health emergency response capability for primary health institutions.

First-level Indicators	Second-level Indicators	Third-level Indicators
(A) Prevention and monitoring	(A_1_) Health management and education for key populations	(A_11_) Frequency of real-time push and promotion of emergency-related health knowledge among residents through multiple media
		(A_12_)Health education and health monitoring for key populations
		(A_13_) Educate and instruct service personnel regarding proper cleaning and disinfection and air purification
	(A_2_) Risk assessment and monitoring	(A_21_) Regularly conduct regional risk identification and judgment
		(A_22_) Fever sentinel
(B) Resource reserve and system building	(B_1_) Human resources	(B_11_) Degree/education of the emergency response team
		(B_12_) Rating the completeness of specialized emergency response team set-up
		(B_13_) Turnover rate of emergency public health personnel
	(B_2_) Material resources	(B_21_) Establishment of an emergency stockpile catalog and emergency procurement plan
		(B_22_) A management system for deploying emergency supplies and equipment
		(B_23_) The renewal rate of emergency supplies
	(B_3_) Management system	(B_31_) Emergency leadership team
		(B_32_) Permanent emergency management department/section
		(B_33_) Emergency duty system
		(B_34_) Emergency file management system
		(B_35_) Sectoral division of labor and communication mechanisms in emergency conditions
		(B_36_) Delineate the responsibilities of the emergency response team
	(B_4_) Contingency plan for prevention and control	(B_41_) How many emergency response plans exist for public health emergencies
		(B_42_) Frequency of revising the plan
	(B_5_) Emergency response training and exercises	(B_51_) Emergency response training for new employees
		(B_52_) Average content and frequency of annual training in emergency response skills
		(B_53_) Average number of annual emergency response simulation drills organized by the department in response to emergency conditions
		(B_54_) Average annual number of participants in emergency response drills at the district level and above
		(B_55_) Pass rate of the most recent emergency drill test for healthcare workers
(C) Emergency response and summarization	(C_1_) Emergency communication and information reporting	(C_11_) Report management process
		(C_12_) Clarification of reporting lines of authority and accountability of responsible departments and individuals
	(C_2_) Patient care and transportation	(C_21_) Establishment of an emergency treatment guideline and management mechanism for community health service centers
		(C_22_) Areas of isolation and protection against infectious diseases and corresponding measures
		(C_23_) Pre-screening and triage table
		(C_24_) Whether the green channel is effectively open
		(C_25_) Provision of basic medical and preventive services to persons under intensive or home-based medical observation
		(C_26_) Robust patient transfer and classification mechanisms
	(C_3_) Epidemiological surveys	(C_31_) Epidemiological survey system
		(C_32_) Number of emergency response team personnel who conducted epidemiological surveys within the past year
	(C_4_) Emergency response summary	(C_41_) Conducting case-by-case assessments of public health emergencies
		(C_42_) Determining incentives and penalties for health emergency responders
		(C_43_) Keep summary reports of public health emergencies

### 3.6 Determining the weight of each indicator

The final weights of the indicators at all levels are shown in Table 3. The CR of each modified judgment matrix was lower than 0.10, the consistency of the judgment matrix was good, and the results were scientific and credible. The weight analysis revealed that “Emergency Response and Summary” constituted the highest proportion (0.379) among primary indicators, followed by “Resource Reserve and System Construction” (0.335) and “Prevention and Monitoring” (0.286). At the secondary indicator level, the top three weighted components were “Risk Assessment and Monitoring” (0.167), “Patient Care and Transportation” (0.144), and “Health Management and Education for Key Populations” (0.119).

**Table 3:** The weight values of the three-level indicator system.

First-level indicators	Weight	Second-level indicators	Weight	Combined weight	Third-level indicators	Weight	Combined weight
A	0.286	A_1_	0.417	0.119	A_11_	0.286	0.034
					A_12_	0.426	0.051
					A_13_	0.288	0.034
		A_2_	0.583	0.167	A_21_	0.431	0.072
					A_22_	0.569	0.095
B	0.335	B_1_	0.253	0.085	B_11_	0.148	0.013
					B_12_	0.551	0.047
					B_13_	0.301	0.025
		B_2_	0.212	0.071	B_21_	0.365	0.026
					B_22_	0.311	0.022
					B_23_	0.324	0.023
		B_3_	0.138	0.046	B_31_	0.233	0.011
					B_32_	0.167	0.008
					B_33_	0.139	0.006
					B_34_	0.095	0.004
					B_35_	0.198	0.009
					B_36_	0.168	0.008
		B_4_	0.195	0.065	B_41_	0.569	0.037
					B_42_	0.431	0.028
		B_5_	0.202	0.068	B_51_	0.183	0.013
					B_52_	0.181	0.012
					B_53_	0.196	0.013
					B_54_	0.189	0.013
					B_55_	0.251	0.017
C	0.379	C_1_	0.288	0.109	C_11_	0.584	0.064
					C_12_	0.417	0.045
		C_2_	0.380	0.144	C_21_	0.145	0.021
					C_22_	0.199	0.029
					C_23_	0.147	0.021
					C_24_	0.151	0.022
					C_25_	0.171	0.024
					C_26_	0.187	0.027
		C_3_	0.210	0.080	C_31_	0.492	0.039
					C_32_	0.508	0.041
		C_4_	0.122	0.046	C_41_	0.431	0.020
					C_42_	0.201	0.009
					C_43_	0.368	0.017

## 4 Discussion

To address the potential issues of public health emergencies, it is imperative to establish a scientific public health emergency response system and enhance health emergency response capabilities. Currently, the majority of the studies on the framework of health emergency response capability follow the CDC recommendation as the primary assessment object, adopting a standardized evaluation framework. However, there are few studies on health institutions at all levels, and there is a desperate need for evaluation frameworks for similar types of institutions. Primary health institutions, as the main health management bodies with the closest contact with the population. They are responsible for early detection, prevention, and treatment of patients and public health education during outbreaks. Therefore, it is imperative to evaluate and improve the emergency response capacity of these institutions. Using the Delphi method and AHP, this study constructed a framework to evaluate the health emergency response capability of primary health institutions in metropolis. Tailored to address the unique emergency health needs and characteristics of primary health institutions, the finalized framework comprises 3 first-level indicators, 11 s-level indicators, and 37 third-level indicators. The weights of indicators at each level can delineate the primary focus areas of the health emergency response capacities in primary health institutions. Divergent demands for primary health emergency response capacities exist across regions, such as rural areas and international settings. Future research should conduct systematic validation studies within target regions. Leveraging empirical data to refine indicator weighting and assessment dimensions will enable localized adaptation and optimization of the evaluation system. Other healthcare facilities can also benefit from this study by gaining a deeper understanding of the functions of primary healthcare institutions, thereby enhancing overall emergency response efficiency.

The indicator system constructed in this study employed a combination of the Delphi method and Analytic Hierarchy Process (AHP). Through multiple rounds of feedback and information exchange, expert opinions were integrated, and complex issues were decomposed into various levels and factors based on extensive expert consultation. This allowed for a sequential evaluation of the relative importance of each indicator. This multi-criteria decision-making approach, which combined quantitative and qualitative methods, significantly enhanced the rigor and credibility of the indicator system.

The indicator system developed in this study significantly differs from the indicator systems of other countries. The United States’ National Health Security Preparedness Index (22) (NHSPI) and Europe’s Health Emergency Preparedness Self-Assessment Tool (23) (HEPSA) are national or state-level systems. Responsibility for achieving these capabilities spans across both public and private sector agencies and organizations, from federal, state, and local public health and emergency management to health care providers, businesses, and volunteer organizations. In contrast, China’s “Health Emergency Capability Assessment Survey Questionnaire” (10) is designed to assess the public health emergency response capabilities of provincial and municipal health authorities. In this study, the focus of the indicator system was on primary health institutions, with a more specific target and indicators that were more closely aligned with the functions of these institutions during public health emergencies. Furthermore, the NHSPI uses only objective data for evaluation, while both Europe and China combine subjective and objective indicators to assess emergency response capabilities. This study adopted the latter strategy. As primary health institutions continue to develop information technology, more objective indicators can be incorporated, thereby enhancing the validity and authenticity of the indicator system.

This study followed a similar approach to the first-level indicators of HEPSA (23), primarily based on the PPRR (Prevention, Preparedness, Response, Recovery) theory. It divided emergency response to public health emergencies into four stages: prevention, preparedness, response, and recovery. Certain dimensional indicators were combined due to the limited functions of primary health institutions. Among the first-level indicators, “emergency response and summarization” held a weight of 0.379, “resource reserve and system building” held a weight of 0.335, and “prevention and monitoring” held a weight of 0.286. This study showed that “emergency response and summarization” is the most important dimension in evaluating the emergency response capacity of primary health institutions during public health emergencies. Primary health institutions, as organizations that are most likely to contact patients in the first place, are known as the “first on-site responders” in public health emergencies. As core institutions in the joint prevention and control effort during public health emergencies, primary health institutions should promptly detect and track patient contact to strengthen isolation and prevent further transmission (24). Under China’s hierarchical diagnosis and treatment system, proficient patient management and referral services by primary health institutions can significantly reduce the frequency of patient visits at higher-level hospitals, and simultaneously decrease the risk of large-scale cross-infection. “Resource reserve and system building” emerged as the second important dimension. In the early stages of public health emergencies, the shortage of protective equipment and testing services is a common problem, particularly in primary health institutions. Furthermore, a study on Madrid community hospitals during the COVID-19 pandemic showed a marked increase in the need for both non-ICU and ICU beds compared to the pre-pandemic period. Given the limited clinical supplies and available space in primary health institutions, rational emergency plans and response systems are essential (24–29). The dimension of “prevention and monitoring” was given less weight but remained significant. In the crisis prevention and preparedness phase, primary health institutions play a major role in health education, vaccination, and risk surveillance. Taking COVID-19 as an example of health education, a cross-sectional study conducted in Saudi Arabia (2) demonstrated a negative correlation between personal health literacy level and fear of COVID-19. Similarly, a Canadian study (30) reported that educational attainment is associated with vaccination hesitancy. Pétré et al. (31) proposed a detailed patient education strategy to triage patients with COVID-19. It was found that health education provided by primary health institutions can help improve health literacy, eliminate fear regarding disease and vaccines, and spread proper preventive measures among regional residents. In terms of vaccination, a case–control study published in BMJ demonstrated the effectiveness of two COVID-19 vaccines (32). However, due to issues such as inequity in vaccine access and vaccine hesitancy (33–35), community-based vaccination programs are still needed. Moreover, the risk monitoring program conducted by primary health institutions facilitates early detection of public health emergencies and swift response to such emergencies. This proactive approach provides time for subsequent emergency responses.

Among the second-level indicators, “risk assessment and monitoring” and “human resources” were particularly important. The secondary indicator “risk assessment and monitoring” in this study corresponded to the first-level indicators “Support capacity: Surveillance” and “Support capacity: Risk assessment” in HEPSA (23), and to “Health Security Surveillance” and “Incident and Information Management” in NHSPI (22). This highlighted the importance of this indicator in the assessments of emergency response capability. However, since the main responsibility for “risk assessment and monitoring” rested with CDC at various levels, and primary health institutions primarily played a role in the early stages of risk assessment and monitoring, it was regarded as a secondary indicator for assessing the emergency response capabilities of primary health institutions. The public health surveillance system serves as an early warning system during public health emergencies (36) and provides essential scientific evidence for public health decision-making and control measures. Primary health institutions are primarily responsible for real-time monitoring of factors affecting public health emergencies. As part of the community health service centers, fever sentinel sites serve two purposes. They alleviate the pressure on general hospitals and enhance the early detection of patients and public health emergencies. A study conducted in Shenzhen, China showed that utilizing primary health institutions as alternatives to fever clinics in general hospitals improved care accessibility and preserved high-quality healthcare resources (37). Singapore’s Public Health Preventive Clinic system is similar to fever sentinel sites. In this system, community-based general practice clinics work following uniform standards issued by the health department, thereby preserving public health resources (38). The “fever sentinel point” emerged as the most heavily weighted indicator among the three levels within the dimension, reflecting its importance among the experts.

Human resources are the first and strategic resources of primary health institutions, and how to maximize the value of human resources is critical in public health emergencies. The first-level indicator “Resources trained workforce” in HEPSA (23) primarily focused on the capacity and training of public health personnel. In the NHSPI (22). Human resources were not listed as a separate dimension, but were instead dispersed across various dimensions as indicators for assessing the capabilities of emergency response personnel. In this study, human resources were included as a secondary indicator under the category of resource reserves and institutional development to assess the capacity and stability of the public health workforce. On the one hand, human resources are the most valuable resources, which cannot be “urgently manufactured” and used at maximum capacity for a long time (39). During the COVID-19 pandemic, studies were published in the United States (40), India (41), Singapore (42) about human resource shortages, including shortages in key occupational groups and skills (43). In addition, during the outbreak, the working hours of frontline health workers were increased to varying degrees, particularly in Germany and Sweden, where working hours were increased to 40–68 h per week. Finland and Italy legislated for the mandatory recruitment of all health workers between the ages of 18 and 68 (44). Prolonged working hours can encourage frontline health workers to leave their jobs (45, 46), further deteriorating the scarcity of human resources. On the other hand, healthcare workers are at both physical and psychological risk. According to the WHO report, healthcare workers accounted for 8% of global COVID-19 cases, and their risk of infection was three times more than that of the general population (47). High-intensity work also affects the mental health of healthcare workers. A meta-analysis of psychiatric problems among Chinese healthcare workers showed that the prevalence of depression, anxiety, and sleep problems, respectively, accounted for 29, 27, and 40% of all problems among Chinese healthcare workers during the COVID-19 pandemic (48). Similarly, 24.7% of Spanish healthcare workers reported symptoms of acute stress, and 53.6% reported symptoms associated with poor general health (49). An Australian cross-sectional study of primary care nurses showed that 39.6% of participants were experiencing symptoms of depression, anxiety, or stress, and the majority of participants felt that their symptoms were related to COVID-19 (50). These psychological problems may be related to mental stress, physical exhaustion, separation from family, stigmatization, and other mental health-related stressors. Therefore, to maximize the value of human resources in public health emergencies, it is necessary to build a health emergency response team rationally, deploy human resources, and protect the mental health of healthcare workers.

This study had several limitations. Firstly, the developed evaluation framework remains theoretical, lacking empirical validation. However, it is also worth noting that the evaluation framework will be used to evaluate the capacity of primary health institutions in some regions of Shanghai. We will collect and analyze data on primary emergency response capacity in this region. Secondly, this study primarily aimed to develop a comprehensive indicator system for emergency applications in primary health institutions. The existing evaluation framework, developed by an expert panel comprising representatives from community health service centers, CDCs, universities, and the National Health Commission, largely met the assessment requirements for evaluating the public health emergency response capabilities of primary health institutions. However, due to constraints in time, resources, and funding, we could not incorporate the demand for health services and the views of other stakeholders, such as legislators and other health emergency response forces (such as public health centers). Subsequent research incorporating these stakeholders would integrate multidimensional perspectives of policy-making, legal norms, and practical operations, thereby facilitating optimization of the indicator system’s responsibility allocation mechanism to enhance its operational feasibility and scientific rigor. Thirdly, the weights of the indicators depended on the subjective judgment of experts, which might not accurately reflect the preferences of policy-makers. The assessment process must be repeated whenever the structure and participants of the AHP change. In addition, the results will change accordingly, with some subjective effects. To address this issue, future improvements can focus on two dimensions. First, the quality of expert assessments is improved by diversifying the professional backgrounds of the evaluation panel, fostering consensus among experts, and conducting rigorous consistency checks (CR < 0.1). Second, a hybrid weighting model is proposed that combines AHP with fuzzy comprehensive evaluation and entropy methods, effectively balancing subjective expertise with objective data, thereby minimizing bias and enhancing the scientific validity of the results.

## 5 Conclusion

Primary health institutions, as the “first on-site responders,” are responsible for the early detection, prevention, and treatment of patients and public health education. They play a crucial role in responding to health emergencies. Therefore, a sound and effective emergency response system of primary health institutions can protect residents against public health emergencies. Using the Delphi process and AHP, we developed a framework for evaluating the health emergency response capabilities of primary health institutions, comprising 3 first-level indicators, 11 s-level indicators, and 37 third-level indicators. This study provides an objective and practical tool to evaluate the health emergency response capabilities of primary health institutions in the metropolis and help improve the evaluation systems for emergency response capacity in Chinese primary health institutions. The evaluation system constructed in this study holds significant practical value in identifying weaknesses in institutional emergency response capabilities, guiding future capacity-building directions, and optimizing the allocation of health resources. It can markedly enhance the emergency response effectiveness of primary health institutions during public health emergencies. However, the current research has several limitations, including a lack of empirical study support, insufficient incorporation of perspectives from key stakeholders, and potential subjective bias risks associated with the Delphi method and Analytic Hierarchy Process. Future research will focus on conducting systematic empirical analyses, expanding sample coverage, and refining research methodologies to continuously improve the scientific rigor and generalizability of the indicator system. This will provide more reliable evidence-based support for strengthening the emergency response capacity of primary health institutions.

## Data Availability

The original contributions presented in the study are included in the article/Supplementary material, further inquiries can be directed to the corresponding author/s.
