# Supplementary material for: Development of health emergency response capability evaluation framework for primary health institutions in metropolis: based on Delphi method and analytic hierarchy process
Source: Front Public Health. 2025 Jul 23;13:1577853. doi: 10.3389/fpubh.2025.1577853 (PMC12325185; doi:10.3389/fpubh.2025.1577853)
Supplement: Supplementary file 1 [file Data_Sheet_1.docx]

Supplementary Material 1

# Supplementary Figures and Tables

Table S1:Basis for judgments and related assignment of scores

Table S2:Results of expert authoritative coefficient

Table S3:Importance scores for first-level indicators in the first round

Table S4:Importance scores for second-level indicators in the first round

Table S5:Importance scores for third-level indicators in the first round

Table S6: Feasibility scores for first-level indicators in the first round

Table S7: Feasibility scores for second-level indicators in the first round

Table S8: Feasibility scores for third-level indicators in the first round

Table S9:Table of threshold values for the first round of importance scores

Table S10:Table of threshold values for the first round of feasibility scores

Table S11:Importance scores for first-level indicators in the second round

Table S1. Basis for judgments and related assignment of scores

| Basis of judgments | The degree of impact on experts’ judgment | | |
| --- | --- | --- | --- |
|  | Small impact | Medium impact | Significant impact |
| practical experience | 0.30 | 0.40 | 0.50 |
| theoretical analysis | 0.10 | 0.20 | 0.30 |
| reference literature at home and abroad | 0.05 | 0.10 | 0.10 |
| intuitive feeling | 0.05 | 0.10 | 0.10 |

Table S2. Results of expert authoritative coefficient

| Advisory rounds | C_a_ | C_s_ | C_r_ |
| --- | --- | --- | --- |
| first round | 0.93 | 0.91 | 0.92 |
| second round | 0.91 | 0.93 | 0.92 |

Table S3. Importance scores for first-level indicators in the first round

| Indicator content | Mean score | Standard deviation | percentage of full scores | variation coefficients |
| --- | --- | --- | --- | --- |
| Prevention and monitoring | 4.867 | 0.352 | 0.867 | 0.072 |
| resource reserve and system building | 4.933 | 0.258 | 0.933 | 0.052 |
| response and Summarization | 4.933 | 0.258 | 0.933 | 0.052 |

Table S4. Importance scores for second-level indicators in the first round

| Indicator content | Mean score | Standard deviation | percentage of full scores | variation coefficients |
| --- | --- | --- | --- | --- |
| Health management and education for key populations | 4.800 | 0.414 | 0.800 | 0.086 |
| Risk assessment and early warning monitoring | 4.800 | 0.414 | 0.800 | 0.086 |
| human resources | 4.933 | 0.258 | 0.933 | 0.052 |
| material resources | 4.933 | 0.258 | 0.933 | 0.052 |
| Management system | 4.867 | 0.352 | 0.867 | 0.072 |
| Organizational division of labor | 4.933 | 0.258 | 0.933 | 0.052 |
| Contingency plan for prevention and control | 4.933 | 0.258 | 0.933 | 0.052 |
| Emergency Response Training and Exercises | 5.000 | 0 | 1.000 | 0 |
| Emergency communication and reporting | 4.933 | 0.258 | 0.933 | 0.0523 |
| Patient care and transportation | 4.933 | 0.258 | 0.933 | 0.052 |
| Summary and evaluation | 4.867 | 0.516 | 0.933 | 0.106 |

Table S5. Importance scores for third-level indicators in the first round

| Indicator content | Mean score | Standard deviation | percentage of full scores | variation coefficients |
| --- | --- | --- | --- | --- |
| Frequency of real-time push and promotion of emergency-related health knowledge to residents in the district through multiple communication media | 4.867 | 0.352 | 0.867 | 0.072 |
| Health education, health monitoring and vaccination of key populations | 4.933 | 0.258 | 0.933 | 0.052 |
| Educate and instruct service personnel on proper cleaning and disinfection and air purification | 4.800 | 0.414 | 0.800 | 0.086 |
| Regularly conduct regional risk identification and judgment | 4.600 | 0.632 | 0.667 | 0.137 |
| Development of norms for categorized medical records | 4.533 | 1.302 | 0.800 | 0.287 |
| Number of public health emergencies in the past year | 4.333 | 1.175 | 0.667 | 0.271 |
| Incidence of class A, B and C infectious diseases | 4.533 | 0.743 | 0.667 | 0.164 |
| Organization of monitoring of "three small places" | 3.733 | 1.668 | 0.400 | 0.447 |
| fever sentinel | 4.933 | 0.258 | 0.933 | 0.052 |
| Development of an epidemiological investigation system with specialized personnel | 5.000 | 0 | 1.000 | 0 |
| Pool of emergency response experts | 4.600 | 0.737 | 0.733 | 0.160 |
| Degree/education composition of the emergency response team | 4.733 | 0.594 | 0.800 | 0.125 |
| Composition of technical titles of emergency response team personnel | 4.200 | 0.941 | 0.533 | 0.224 |
| Rate of completeness of specialized emergency response team set-up | 4.467 | 0.915 | 0.733 | 0.205 |
| Turnover rate of emergency public health workforce personnel | 4.667 | 0.617 | 0.733 | 0.132 |
| Emergency supplies and equipment deployment management system | 4.467 | 1.125 | 0.733 | 0.252 |
| Establishment of an emergency stockpile catalog and emergency procurement plan | 4.800 | 0.561 | 0.867 | 0.117 |
| Establishment of an information management network for emergencies | 4.333 | 0.724 | 0.467 | 0.167 |
| Community Emergency Equipment Reserve | 3.800 | 1.568 | 0.467 | 0.413 |
| Emergency supplies renewal rate | 4.000 | 1.414 | 0.533 | 0.354 |
| Emergency leadership team | 4.933 | 0.258 | 0.933 | 0.052 |
| Permanent emergency management department/section | 4.667 | 0.617 | 0.733 | 0.132 |
| Emergency duty system | 4.800 | 0.561 | 0.867 | 0.117 |
| Emergency file management system | 4.667 | 0.617 | 0.733 | 0.132 |
| Sectoral division of labor and communication mechanisms in times of emergency | 4.733 | 0.594 | 0.800 | 0.125 |
| Delineate the responsibilities of the emergency response team | 4.800 | 0.561 | 0.867 | 0.117 |
| Whether there is an emergency response plan for public health emergencies, and the number of such plans | 4.733 | 0.704 | 0.867 | 0.149 |
| Frequency of revision of the plan | 4.533 | 0.743 | 0.667 | 0.164 |
| Emergency response training for new recruits | 4.867 | 0.516 | 0.933 | 0.106 |
| Average annual content and frequency of training in emergency response skills | 4.800 | 0.561 | 0.867 | 0.117 |
| Annual average number of emergency response simulation drills organized by the department in response to emergencies | 4.667 | 0.724 | 0.800 | 0.155 |
| Average annual number of participants in emergency response drills at the district level and above | 4.067 | 1.438 | 0.600 | 0.354 |
| Pass rate of the most recent emergency drill test for health care workers | 4.867 | 0.516 | 0.933 | 0.106 |
| Report management process | 5.000 | 0 | 1.000 | 0 |
| Clarification of reporting lines of authority and accountability of responsible departments and individuals. | 5.000 | 0 | 1.000 | 0 |
| Establishment of an emergency treatment guidance and management mechanism with community health service stations under its jurisdiction | 4.667 | 0.617 | 0.733 | 0.132 |
| Areas of isolation and protection against infectious diseases and corresponding measures | 5.000 | 0 | 1.000 | 0 |
| pre-screening and triage table | 4.933 | 0.2582 | 0.933 | 0.052 |
| Whether the green channel is effectively open | 4.867 | 0.516 | 0.933 | 0.106 |
| Provision of basic medical and preventive services to persons under intensive or home-based medical observation | 4.867 | 0.352 | 0.867 | 0.072 |
| Standby emergency vaccinations and prophylactic medications | 4.867 | 0.352 | 0.867 | 0.072 |
| Robust patient transfer and diversion mechanisms | 4.733 | 0.704 | 0.867 | 0.149 |
| Conducting case-by-case assessments of public health emergencies | 4.667 | 0.724 | 0.800 | 0.155 |
| Developing incentives and penalties for health emergency responders | 4.733 | 0.594 | 0.800 | 0.125 |
| Keep summary reports of public health emergencies | 4.667 | 0.617 | 0.733 | 0.132 |

Table S6. Feasibility scores for first-level indicators in the first round

| Indicator content | Mean score | Standard deviation | percentage of full scores | variation coefficients |
| --- | --- | --- | --- | --- |
| Prevention and monitoring | 4.733 | 0.594 | 0.800 | 0.125 |
| resource reserve and system building | 4.867 | 0.352 | 0.867 | 0.072 |
| response and Summarization | 4.733 | 0.594 | 0.800 | 0.125 |

Table S7. Feasibility scores for second-level indicators in the first round

| Indicator content | Mean score | Standard deviation | percentage of full scores | variation coefficients |
| --- | --- | --- | --- | --- |
| Health management and education for key populations | 4.733 | 0.594 | 0.800 | 0.125 |
| Risk assessment and early warning monitoring | 4.333 | 1.047 | 0.533 | 0.242 |
| human resources | 4.733 | 0.594 | 0.800 | 0.125 |
| material resources | 4.933 | 0.258 | 0.933 | 0.052 |
| Management system | 4.800 | 0.414 | 0.800 | 0.086 |
| Organizational division of labor | 4.867 | 0.352 | 0.867 | 0.072 |
| Contingency plan for prevention and control | 4.933 | 0.258 | 0.933 | 0.052 |
| Emergency Response Training and Exercises | 4.867 | 0.352 | 0.867 | 0.072 |
| Emergency communication and reporting | 5.000 | 0 | 1.000 | 0 |
| Patient care and transportation | 4.400 | 0.828 | 0.600 | 0.188 |
| Summary and evaluation | 4.933 | 0.258 | 0.933 | 0.052 |

Table S8. Feasibility scores for third-level indicators in the first round

| Indicator content | Mean score | Standard deviation | percentage of full scores | variation coefficients |
| --- | --- | --- | --- | --- |
| Frequency of real-time push and promotion of emergency-related health knowledge to residents in the district through multiple communication media | 4.867 | 0.352 | 0.867 | 0.072 |
| Health education, health monitoring and vaccination of key populations | 4.600 | 0.507 | 0.600 | 0.110 |
| Educate and instruct service personnel on proper cleaning and disinfection and air purification | 4.400 | 0.828 | 0.600 | 0.188 |
| Regularly conduct regional risk identification and judgment | 4.400 | 0.828 | 0.600 | 0.188 |
| Development of norms for categorized medical records | 4.533 | 1.302 | 0.800 | 0.287 |
| Number of public health emergencies in the past year | 4.667 | 0.724 | 0.800 | 0.155 |
| Incidence of class A, B and C infectious diseases | 4.600 | 0.737 | 0.733 | 0.160 |
| Organization of monitoring of "three small places" | 3.533 | 1.598 | 0.267 | 0.452 |
| fever sentinel | 4.933 | 0.258 | 0.933 | 0.052 |
| Development of an epidemiological investigation system with specialized personnel | 4.800 | 0.561 | 0.867 | 0.117 |
| Pool of emergency response experts | 4.333 | 1.047 | 0.667 | 0.242 |
| Degree/education composition of the emergency response team | 4.267 | 0.961 | 0.533 | 0.225 |
| Composition of technical titles of emergency response team personnel | 4.067 | 1.100 | 0.533 | 0.270 |
| Rate of completeness of specialized emergency response team set-up | 4.067 | 1.033 | 0.467 | 0.254 |
| Turnover rate of emergency public health workforce personnel | 3.933 | 1.100 | 0.400 | 0.280 |
| Emergency supplies and equipment deployment management system | 4.400 | 1.183 | 0.733 | 0.269 |
| Establishment of an emergency stockpile catalog and emergency procurement plan | 4.600 | 0.910 | 0.800 | 0.198 |
| Establishment of an information management network for emergencies | 3.867 | 0.990 | 0.333 | 0.256 |
| Community Emergency Equipment Reserve | 3.733 | 1.624 | 0.467 | 0.435 |
| Emergency supplies renewal rate | 3.467 | 1.552 | 0.333 | 0.448 |
| Emergency leadership team | 4.933 | 0.258 | 0.933 | 0.052 |
| Permanent emergency management department/section | 4.400 | 0.986 | 0.667 | 0.224 |
| Emergency duty system | 4.467 | 1.125 | 0.733 | 0.252 |
| Emergency file management system | 4.600 | 0.632 | 0.667 | 0.137 |
| Sectoral division of labor and communication mechanisms in times of emergency | 4.600 | 0.737 | 0.733 | 0.160 |
| Delineate the responsibilities of the emergency response team | 4.600 | 0.737 | 0.733 | 0.160 |
| Whether there is an emergency response plan for public health emergencies, and the number of such plans | 4.533 | 0.834 | 0.733 | 0.184 |
| Frequency of revision of the plan | 4.467 | 0.743 | 0.600 | 0.166 |
| Emergency response training for new recruits | 4.733 | 0.594 | 0.800 | 0.125 |
| Average annual content and frequency of training in emergency response skills | 4.800 | 0.561 | 0.867 | 0.117 |
| Annual average number of emergency response simulation drills organized by the department in response to emergencies | 4.733 | 0.704 | 0.867 | 0.149 |
| Average annual number of participants in emergency response drills at the district level and above | 4.200 | 1.207 | 0.600 | 0.287 |
| Pass rate of the most recent emergency drill test for health care workers | 4.667 | 0.617 | 0.733 | 0.132 |
| Report management process | 4.667 | 0.724 | 0.800 | 0.155 |
| Clarification of reporting lines of authority and accountability of responsible departments and individuals. | 4.867 | 0.352 | 0.867 | 0.072 |
| Establishment of an emergency treatment guidance and management mechanism with community health service stations under its jurisdiction | 4.600 | 0.737 | 0.733 | 0.160 |
| Areas of isolation and protection against infectious diseases and corresponding measures | 4.800 | 0.561 | 0.867 | 0.117 |
| pre-screening and triage table | 4.733 | 1.033 | 0.933 | 0.218 |
| Whether the green channel is effectively open | 4.733 | 0.799 | 0.867 | 0.169 |
| Provision of basic medical and preventive services to persons under intensive or home-based medical observation | 4.733 | 0.799 | 0.867 | 0.169 |
| Standby emergency vaccinations and prophylactic medications | 4.733 | 0.594 | 0.800 | 0.125 |
| Robust patient transfer and diversion mechanisms | 4.667 | 0.724 | 0.800 | 0.155 |
| Conducting case-by-case assessments of public health emergencies | 4.400 | 0.986 | 0.667 | 0.224 |
| Developing incentives and penalties for health emergency responders | 4.600 | 0.910 | 0.800 | 0.198 |
| Keep summary reports of public health emergencies | 4.600 | 0.632 | 0.667 | 0.137 |

Table S9. Table of threshold values for the first round of importance scores

|  | Mean score | Standard deviation | cut-off values |
| --- | --- | --- | --- |
| Mean value | 4.714 | 0.287 | 4.427 |
| Variation coefficients | 0.125 | 0.097 | 0.222 |
| Percentage of perfect scores | 0.817 | 0.143 | 0.674 |

Table S10. Table of threshold values for the first round of feasibility scores

|  | Mean score | Standard deviation | cut-off values |
| --- | --- | --- | --- |
| Mean value | 4.556 | 0.341 | 4.215 |
| Variation coefficients | 0.171 | 0.094 | 0.265 |
| Percentage of perfect scores | 0.732 | 0.166 | 0.566 |

Table S11. Importance scores for first-level indicators in the second round

| Indicator content | Mean value | Standard deviation | percentage of full scores | variation coefficients |
| --- | --- | --- | --- | --- |
| Prevention and monitoring | 4.933 | 0.258 | 0.933 | 0.052 |
| resource reserve and system building | 5.000 | 0 | 1.000 | 0 |
| Emergency Response and Disposal | 4.933 | 0.258 | 0.933 | 0.052 |
| Summary and evaluation | 4.800 | 0.561 | 0.867 | 0.117 |
