# Supplementary material for: Development of health emergency response capability evaluation framework for primary health institutions in metropolis: based on Delphi method and analytic hierarchy process
Source: Front Public Health. 2025 Jul 23;13:1577853. doi: 10.3389/fpubh.2025.1577853 (PMC12325185; doi:10.3389/fpubh.2025.1577853)
Supplement: Supplementary file 2 [file Data_Sheet_2.docx]

Supplementary Material 2

# Supplementary Figures and Tables

Table S12:Importance scores for second-level indicators in the second round

Table S13:Importance scores for third-level indicators in the second round

Table S14:Feasibility scores for first-level indicators in the second round

Table S15:Feasibility scores for second-level indicators in the second round

Table S16:Feasibility scores for third-level indicators in the second round

Table S17:Table of threshold values for the second round of importance scores

Table S18:Table of threshold values for the second round of feasibility scores

Table S19. Table of Explanation and Scoring Criteria for Tertiary Indicators

Table S12. Importance scores for second-level indicators in the second round

| Indicator content | Mean score | Standard deviation | percentage of full scores | variation coefficients |
| --- | --- | --- | --- | --- |
| Health management and education for key populations | 4.867 | 0.351 | 0.867 | 0.072 |
| Risk assessment and monitoring | 4.800 | 0.414 | 0.800 | 0.086 |
| human resources | 5.000 | 0 | 1.000 | 0 |
| material resources | 4.933 | 0.258 | 0.933 | 0.052 |
| Management system | 4.933 | 0.258 | 0.933 | 0.052 |
| Contingency plan for prevention and control | 5.000 | 0 | 1.000 | 0 |
| Emergency Response Training and Exercises | 5.000 | 0 | 1.000 | 0 |
| Emergency communication and information reporting | 5.000 | 0 | 1.000 | 0 |
| Patient care and transportation | 5.000 | 0 | 1.000 | 0 |
| Epidemiological surveys | 4.933 | 0.258 | 0.933 | 0.052 |
| Evaluation of major event security | 4.600 | 1.056 | 0.800 | 0.229 |
| Emergency Response Summary | 4.933 | 0.258 | 0.933 | 0.052 |

Table S13. Importance scores for third-level indicators in the second round

| Indicator content | Mean score | Standard deviation | percentage of full scores | variation coefficients |
| --- | --- | --- | --- | --- |
| Frequency of real-time push and promotion of emergency-related health knowledge to residents in the district through multiple communication media | 4.800 | 0.561 | 0.867 | 0.117 |
| Health education, health monitoring and vaccination of key populations | 5.000 | 0 | 1.000 | 0 |
| Educate and instruct service personnel on proper cleaning and disinfection and air purification | 4.800 | 0.414 | 0.800 | 0.086 |
| Regularly conduct regional risk identification and judgment | 4.733 | 0.458 | 0.733 | 0.097 |
| fever sentinel | 5.000 | 0 | 1.000 | 0 |
| Pool of emergency response experts | 4.400 | 1.183 | 0.733 | 0.269 |
| Degree/education composition of the emergency response team | 4.800 | 0.414 | 0.8 | 0.086 |
| Rate of completeness of specialized emergency response team set-up | 4.600 | 0.737 | 0.733 | 0.160 |
| Turnover rate of emergency public health workforce personnel | 4.600 | 0.632 | 0.667 | 0.137 |
| Establishment of an emergency stockpile catalog and emergency procurement plan | 4.800 | 0.561 | 0.867 | 0.117 |
| Emergency supplies and equipment deployment management system | 4.800 | 0.561 | 0.867 | 0.117 |
| Emergency supplies renewal rate | 4.800 | 0.561 | 0.867 | 0.117 |
| Emergency leadership team | 4.867 | 0.516 | 0.933 | 0.106 |
| Permanent emergency management department/section | 4.733 | 0.704 | 0.867 | 0.147 |
| Emergency duty system | 4.933 | 0.258 | 0.933 | 0.052 |
| Emergency file management system | 4.933 | 0.258 | 0.933 | 0.052 |
| Sectoral division of labor and communication mechanisms in times of emergency | 4.933 | 0.258 | 0.933 | 0.052 |
| Delineate the responsibilities of the emergency response team | 4.733 | 0.594 | 0.8 | 0.125 |
| Whether there is an emergency response plan for public health emergencies, and the number of such plans | 5.000 | 0 | 1.000 | 0 |
| Frequency of revision of the plan | 4.600 | 0.737 | 0.733 | 0.160 |
| Emergency response training for new recruits | 4.933 | 0.258 | 0.933 | 0.052 |
| Average annual content and frequency of training in emergency response skills | 4.867 | 0.516 | 0.933 | 0.106 |
| Annual average number of emergency response simulation drills organized by the department in response to emergencies | 4.867 | 0.516 | 0.933 | 0.106 |
| Average annual number of participants in emergency response drills at the district level and above | 4.867 | 0.516 | 0.933 | 0.106 |
| Pass rate of the most recent emergency drill test for health care workers | 5.000 | 0 | 1.000 | 0 |
| Report management process | 4.933 | 0.258 | 0.933 | 0.052 |
| Clarification of reporting lines of authority and accountability of responsible departments and individuals. | 4.867 | 0.352 | 0.867 | 0.072 |
| Establishment of an emergency treatment guidance and management mechanism with community health service stations under its jurisdiction | 4.867 | 0.352 | 0.867 | 0.072 |
| Areas of isolation and protection against infectious diseases and corresponding measures | 4.800 | 0.561 | 0.867 | 0.117 |
| pre-screening and triage table | 5.000 | 0 | 1.000 | 0 |
| Whether the green channel is effectively open | 5.000 | 0 | 1.000 | 0 |
| Provision of basic medical and preventive services to persons under intensive or home-based medical observation | 4.867 | 0.516 | 0.933 | 0.106 |
| Robust patient transfer and diversion mechanisms | 4.800 | 0.561 | 0.867 | 0.117 |
| Epidemiological survey system | 5.000 | 0 | 1.000 | 0 |
| Number of emergency response team personnel in the unit who have conducted epidemiological investigations within the past year | 4.733 | 0.594 | 0.800 | 0.125 |
| Participation in major event coverage | 4.467 | 1.125 | 0.733 | 0.252 |
| What level of major event coverage has the unit been involved in? | 4.467 | 1.125 | 0.733 | 0.252 |
| Recognition for relevant activities | 3.200 | 1.699 | 0.267 | 0.531 |
| Conducting case-by-case assessments of public health emergencies | 4.600 | 0.828 | 0.800 | 0.180 |
| Developing incentives and penalties for health emergency responders | 4.667 | 0.617 | 0.733 | 0.132 |
| Keep summary reports of public health emergencies | 4.667 | 0.724 | 0.800 | 0.155 |
| Whether the incidence rate of A, B and C infectious diseases in the area of responsibility in the past three years is higher than the average in the district (prefecture-level city) in which it is located. | 4.000 | 1.464 | 0.600 | 0.366 |

Table S14. Feasibility scores for first-level indicators in the second round

| Indicator content | Mean score | Standard deviation | percentage of full scores | variation coefficients |
| --- | --- | --- | --- | --- |
| Prevention and monitoring | 4.600 | 0.632 | 0.667 | 0.137 |
| resource reserve and system building | 4.933 | 0.258 | 0.933 | 0.052 |
| Emergency Response and Disposal | 4.867 | 0.352 | 0.867 | 0.072 |
| Summary and evaluation | 4.733 | 0.594 | 0.800 | 0.125 |

Table S15. Feasibility scores for second-level indicators in the second round

| Indicator content | Mean score | Standard deviation | percentage of full scores | variation coefficients |
| --- | --- | --- | --- | --- |
| Health management and education for key populations | 4.867 | 0.352 | 0.867 | 0.072 |
| Risk assessment and monitoring | 4.467 | 1.060 | 0.667 | 0.237 |
| human resources | 4.867 | 0.352 | 0.867 | 0.072 |
| material resources | 4.867 | 0.352 | 0.867 | 0.072 |
| Management system | 4.933 | 0.258 | 0.933 | 0.052 |
| Contingency plan for prevention and control | 4.933 | 0.258 | 0.933 | 0.052 |
| Emergency Response Training and Exercises | 5.000 | 0 | 1.000 | 0 |
| Emergency communication and information reporting | 4.933 | 0.258 | 0.933 | 0.052 |
| Patient care and transportation | 5.000 | 0 | 1.000 | 0 |
| Epidemiological surveys | 4.667 | 0.617 | 0.733 | 0.132 |
| Evaluation of major event security | 4.333 | 1.113 | 0.600 | 0.257 |
| Emergency Response Summary | 4.733 | 0.594 | 0.800 | 0.125 |

Table S16. Feasibility scores for third-level indicators in the second round

| Indicator content | Mean score | Standard deviation | percentage of full scores | variation coefficients |
| --- | --- | --- | --- | --- |
| Frequency of real-time push and promotion of emergency-related health knowledge to residents in the district through multiple communication media | 4.800 | 0.414 | 0.800 | 0.086 |
| Health education, health monitoring and vaccination of key populations | 4.867 | 0.352 | 0.867 | 0.072 |
| Educate and instruct service personnel on proper cleaning and disinfection and air purification | 4.533 | 0.743 | 0.667 | 0.164 |
| Regularly conduct regional risk identification and judgment | 4.33 | 0.900 | 0.533 | 0.208 |
| fever sentinel | 4.933 | 0.258 | 0.933 | 0.052 |
| Pool of emergency response experts | 3.867 | 1.246 | 0.400 | 0.322 |
| Degree/education composition of the emergency response team | 4.533 | 0.834 | 0.667 | 0.184 |
| Rate of completeness of specialized emergency response team set-up | 4.467 | 0.915 | 0.667 | 0.205 |
| Turnover rate of emergency public health workforce personnel | 4.400 | 0.910 | 0.600 | 0.207 |
| Establishment of an emergency stockpile catalog and emergency procurement plan | 4.467 | 0.990 | 0.733 | 0.222 |
| Emergency supplies and equipment deployment management system | 4.467 | 0.831 | 0.667 | 0.187 |
| Emergency supplies renewal rate | 4.667 | 0.728 | 0.800 | 0.155 |
| Emergency leadership team | 4.867 | 0.352 | 0.867 | 0.072 |
| Permanent emergency management department/section | 4.533 | 0.990 | 0.800 | 0.218 |
| Emergency duty system | 4.800 | 0.561 | 0.867 | 0.117 |
| Emergency file management system | 4.733 | 0.458 | 0.733 | 0.097 |
| Sectoral division of labor and communication mechanisms in times of emergency | 4.733 | 0.594 | 0.800 | 0.125 |
| Delineate the responsibilities of the emergency response team | 4.533 | 0.743 | 0.667 | 0.164 |
| Whether there is an emergency response plan for public health emergencies, and the number of such plans | 4.800 | 0.561 | 0.867 | 0.117 |
| Frequency of revision of the plan | 4.467 | 0.834 | 0.667 | 0.187 |
| Emergency response training for new recruits | 4.600 | 0.737 | 0.733 | 0.160 |
| Average annual content and frequency of training in emergency response skills | 4.867 | 0.516 | 0.933 | 0.106 |
| Annual average number of emergency response simulation drills organized by the department in response to emergencies | 4.933 | 0.258 | 0.933 | 0.052 |
| Average annual number of participants in emergency response drills at the district level and above | 4.933 | 0.258 | 0.933 | 0.052 |
| Pass rate of the most recent emergency drill test for health care workers | 4.800 | 0.561 | 0.867 | 0.117 |
| Report management process | 4.800 | 0.414 | 0.800 | 0.086 |
| Clarification of reporting lines of authority and accountability of responsible departments and individuals. | 4.733 | 0.594 | 0.800 | 0.125 |
| Establishment of an emergency treatment guidance and management mechanism with community health service stations under its jurisdiction | 4.733 | 0.458 | 0.733 | 0.097 |
| Areas of isolation and protection against infectious diseases and corresponding measures | 4.800 | 0.561 | 0.867 | 0.117 |
| pre-screening and triage table | 4.933 | 0.258 | 0.933 | 0.052 |
| Whether the green channel is effectively open | 4.933 | 0.258 | 0.933 | 0.052 |
| Provision of basic medical and preventive services to persons under intensive or home-based medical observation | 4.733 | 0.594 | 0.800 | 0.125 |
| Robust patient transfer and diversion mechanisms | 4.533 | 0.834 | 0.733 | 0.184 |
| Epidemiological survey system | 4.667 | 0.617 | 0.733 | 0.132 |
| Number of emergency response team personnel in the unit who have conducted epidemiological investigations within the past year | 4.467 | 1.060 | 0.667 | 0.237 |
| Participation in major event coverage | 4.133 | 1.598 | 0.667 | 0.387 |
| What level of major event coverage has the unit been involved in | 3.933 | 1.387 | 0.467 | 0.353 |
| Recognition for relevant activities | 3.333 | 1.589 | 0.267 | 0.477 |
| Conducting case-by-case assessments of public health emergencies | 4.267 | 0.884 | 0.533 | 0.208 |
| Developing incentives and penalties for health emergency responders | 4.400 | 0.737 | 0.533 | 0.167 |
| Keep summary reports of public health emergencies | 4.400 | 0.828 | 0.600 | 0.188 |
| Whether the incidence rate of A, B and C infectious diseases in the area of responsibility in the past three years is higher than the average in the district (prefecture-level city) in which it is located. | 3.800 | 1.656 | 0.600 | 0.436 |

Table S17. Table of threshold values for the second round of importance scores

|  | Mean score | Standard deviation | cut-off values |
| --- | --- | --- | --- |
| Mean value | 4.793 | 0.284 | 4.510 |
| Variation coefficients | 0.099 | 0.096 | 0.195 |
| Percentage of perfect scores | 0.871 | 0.128 | 0.743 |

Table S18. Table of threshold values for the second round of feasibility scores

|  | Mean score | Standard deviation | cut-off values |
| --- | --- | --- | --- |
| Mean value | 4.625 | 0.327 | 4.298 |
| Variation coefficients | 0.148 | 0.099 | 0.247 |
| Percentage of perfect scores | 0.761 | 0.154 | 0.607 |

Table S19. Table of Explanation and Scoring Criteria for Tertiary Indicators

| Third-level Indicators | Explanation and Description of Indicators | Scoring Criteria |
| --- | --- | --- |
| (A_11_) Frequency of real-time push and promotion of emergency-related health knowledge among residents through multiple media | At least include content related to the protection against public health emergencies, self-rescue in earthquake disasters, and first aid for cardiopulmonary resuscitation. | No health knowledge promotion conducted: 0 points;  once to three times a year: 0.5 points;  more than three times a year: 1 point. |
| (A_12_) Health education and health monitoring for key populations | Key populations include the elderly, individuals with chronic underlying conditions, pregnant women, children, persons with disabilities, and individuals with intellectual disabilities. For different types of public health emergencies, appropriate health education and health monitoring should be conducted for these key populations. | Health education and health monitoring for key populations are very comprehensive: 1 point.  Health education and health monitoring for key populations are partial or absent: 0 points. |
| (A_13_) Educate and instruct service personnel regarding proper cleaning and disinfection and air purification | Service personnel in the area need to engage with a large number of people and should focus on promoting infectious disease prevention knowledge, as well as cleaning, disinfection, and air purification | Yes: 1 point. No: 0 points |
| (A_21_) Regularly conduct regional risk identification and judgment | There should be relevant systems or regulations for regularly conducting regional risk identification within the community. | Yes: 1 point. No: 0 points |
| (A_22_) Fever sentinel | This mainly refers to whether the community hospital has set up a fever (sentinel) clinic. | Yes: 1 point. No: 0 points |
| (B_11_) Degree/education of the emergency response team | Number of health technicians with an associate degree or higher / Total number of health technicians during the same period × 100%." | Calculate the coefficient according to the formula |
| (B_12_) Rating the completeness of specialized emergency response team set-up | The emergency team should include personnel responsible for emergency management, response to acute infectious disease outbreaks, emergency medical rescue, and health emergency volunteers. | Missing three or more types of personnel: 0 points;  missing one to three types of personnel: 0.5 points;  complete functions: 1 point |
| (B_13_) Turnover rate of emergency public health personnel | The emergency team includes a standing team and a reserve team. The turnover rate mainly refers to the number of personnel entering and leaving the emergency public health team within a year / Total number of personnel in the emergency public health team at the end of the year | Calculate the coefficient according to the formula |
| (B_21_) Establishment of an emergency stockpile catalog and emergency procurement plan | The unit should establish a catalog of material reserves at this level. The forms of emergency material reserves mainly include physical reserves, commercial reserves, production capacity reserves, and contractual reserves. | No relevant catalog or procurement plan established: 0 points;  catalog and emergency plan established: 0.5 points;  current catalog and procurement plan are the latest version within five years: 1 point |
| (B_22_) A management system for deploying emergency supplies and equipment | The unit should implement complete systems for the procurement, acceptance, storage, issuance, replenishment, updating, and safety of materials listed in the catalog of material reserves at this level. | No relevant systems established: 0 points;  relevant systems established: 0.5 points;  current systems are the latest version within five years: 1 point. |
| (B_23_) The renewal rate of emergency supplies | Verify whether there are personal protective equipment items in the emergency reserve that are within one quarter of their expiration date, and check if commonly used medications and other consumables include any that are within two months of their expiration date | Consumables not updated on time: 0 points;  some consumables updated on time: 0.5 points;  all consumables updated promptly: 1 point. |
| (B_31_) Emergency leadership team | The emergency leadership group is the organization responsible for unified command and coordination during the occurrence of public health emergencies. | No emergency leadership group established: 0 points;  a fixed emergency leadership group: 0.5 points;  emergency leadership groups composed differently based on varying situations: 1 point |
| (B_32_) Permanent emergency management department/section | Community emergency management departments/sections mainly refer to those departments capable of exercising emergency response abilities for public health emergencies, such as the Disease Prevention and Control Section. | No relevant departments or sections established: 0 points;  relevant departments and sections established: 1 point. |
| (B_33_) Emergency duty system | Includes dedicated staff on duty or part-time duty. | No emergency duty system established: 0 points;  relevant system established: 0.5 points;  current system is the latest version within five years: 1 point. |
| (B_34_) Emergency file management system | To standardize emergency document management, ensure that emergency documentation is complete and well-organized, and facilitate retrieval, an emergency document management system has been established. | No relevant system established: 0 points;  relevant system established: 0.5 points;  current system is the latest version within five years: 1 point. |
| (B_35_) Sectoral division of labor and communication mechanisms in emergency conditions | A mechanism for delineating the division of responsibilities and workflows among various departments during emergencies. | No relevant system established: 0 points;  relevant system established: 0.5 points;  current system is the latest version within five years: 1 point. |
| (B_36_) Delineate the responsibilities of the emergency response team | Members of the emergency response team have specific roles, and it is necessary to define the responsibilities of different members during the emergency response process. | No responsibilities defined: 0 points; responsibilities defined: 0.5 points; current system is the latest version within five years: 1 point. |
| (B_41_) How many emergency response plans exist for public health emergencies | The emergency public health plan mainly includes the influenza prevention and control plan, the management plan for outbreaks of infectious diseases, the management plan for unexplained group illnesses, and the plan for handling adverse reactions to vaccinations. | Missing three or more plans: 0 points.  Missing 1 to 3 plans: 0.5 points.  All plans present: 1 point. |
| (B_42_) Frequency of revising the plan | According to the standards, the plans should be revised every two years | Plans not revised for over two years: 0 points;  revised every two years: 0.5 points;  revised within two years based on changes: 1 point. |
| (B_51_) Emergency response training for new employees | New members of the emergency response team need to undergo training, and either training certificates or proof of training arrangements are acceptable (including training programs conducted by various levels of units). | Yes: 1 point. No: 0 points |
| (B_52_) Average content and frequency of annual training in emergency response skills | This mainly includes emergency response skills for various types of emergencies, such as earthquake escape training, cardiopulmonary resuscitation (CPR) training, and so on. | No relevant training: 0 points; training once a year: 0.5 points; training more than once a year: 1 point. |
| (B_53_) Average number of annual emergency response simulation drills organized by the department in response to emergency conditions | This mainly includes emergency response skills for various types of emergencies, such as earthquake evacuation drills, infectious disease outbreak response drills, pest control drills, fire evacuation drills, and so on. | No relevant training: 0 points; training once a year: 0.5 points; training more than once a year: 1 point. |
| (B_54_) Average annual number of participants in emergency response drills at the district level and above | This mainly includes emergency response skills for various types of emergencies, such as earthquake evacuation drills, infectious disease outbreak response drills, pest control drills, fire evacuation drills, and so on. | No relevant training: 0 points; training twice a year: 0.5 points; training more than twice a year: 1 point. |
| (B_55_) Pass rate of the most recent emergency drill test for healthcare workers | The pass rate of the assessment for medical staff after training (based on the most recent emergency drill organized by the district). | Pass rate / 100%. |
| (C_11_) Report management process | To standardize report management work, ensure that reporting materials are complete and well-maintained, and facilitate the retrieval of established reporting management systems. | No relevant system established: 0 points;  relevant system established: 0.5 points;  current system is the latest version within five years: 1 point. |
| (C_12_) Clarification of reporting lines of authority and accountability of responsible departments and individuals | In the reporting process, it is necessary to clarify the reporting rights and responsibilities of each department and the relevant person in charge | Yes: 1 point. No: 0 points |
| (C_21_) Establishment of an emergency treatment guideline and management mechanism for community health service centers | Communities should establish emergency treatment guidance and management mechanisms with their subordinate community health service stations | No relevant system established: 0 points;  relevant system established: 0.5 points;  current system is the latest version within five years: 1 point. |
| (C_22_) Areas of isolation and protection against infectious diseases and corresponding measures | Whether there are relevant systems and rules in the community, and when a public health emergency occurs, an emergency area for isolation and protection of infectious diseases may be divided, and isolation and protection measures may be taken | No relevant system established: 0 points;  relevant system established: 0.5 points;  current system is the latest version within five years: 1 point. |
| (C_23_) Pre-screening and triage table | Community hospitals shall set up Pre-screening and triage table | Yes: 1 point. No: 0 points |
| (C_24_) Whether the green channel is effectively open | Is there a relevant set of regulations in the community to ensure that the green channel can be effectively opened during the occurrence of a public health emergency? | Yes: 1 point. No: 0 points |
| (C_25_) Provision of basic medical and preventive services to persons under intensive or home-based medical observation | Does the community have relevant regulations in place that clearly provide necessary basic medical and preventive services for those under centralized or home medical observation? | Yes: 1 point. No: 0 points |
| (C_26_) Robust patient transfer and classification mechanisms | There should be at least one relatively stable referral hospital that has signed a two-way referral agreement, with traceable referral records, and a two-way referral system established and implemented. | No relevant system established: 0 points;  relevant system established: 0.5 points;  current system is the latest version within five years: 1 point. |
| (C_31_) Epidemiological survey system | To effectively respond to public health emergencies, the community needs to establish relevant systems for epidemiological investigation and allocate personnel accordingly. | Yes: 1 point. No: 0 points |
| (C_32_) Number of emergency response team personnel who conducted epidemiological surveys within the past year | The number of personnel from this unit who participated in epidemiological investigations in the past year. | 0 people participated in epidemiological investigations: 0 points;  3 people participated: 0.5 points;  5 or more people participated: 1 point |
| (C_41_) Conducting case-by-case assessments of public health emergencies | After the public health emergency concludes, conduct case evaluations based on the results of the event to provide experience and recommendations for future work. | No relevant system established: 0 points;  relevant system established: 0.5 points;  current system is the latest version within five years: 1 point. |
| (C_42_) Determining incentives and penalties for health emergency responders | Is there a relevant system or regulation that明确 the rewards and penalties for public health emergency personnel？ | No relevant system established: 0 points;  relevant system established: 0.5 points;  current system is the latest version within five years: 1 point. |
| (C_43_ ) Keep summary reports of public health emergencies | It is required to include at least two reports: an initial report and a case closure report, preferably with progress reports as well, and the reports should contain the basic essential elements. | If the public health summary report for the past month includes only one type of report: 0 points;  if it includes two types of reports: 0.5 points;  if it includes all three types of reports and contains the basic essential elements: 1 point. |
